# Global google trends for construction demonstrate low search volume index for stress, mental and suicide

**DOI:** 10.1186/s13104-023-06628-0

**Published:** 2023-12-19

**Authors:** Craig Steven McLachlan, Hang Truong

**Affiliations:** 1 Centre for Healthy Futures, Torrens University, 3/333 Kent St, Sydney, NSW 2000 Australia; 2 Newcastle Business School, College of Human and Social Futures, University of Newcastle, Callaghan, Australia; 3 Department of International Management Science, Thai Nguyen University of Information and Communication Technology, Thai Nguyen City, 250000 Vietnam

**Keywords:** Construction, Worker, Stress, Suicide, Mental, Search volume index, Google trends

## Abstract

The comparison of Google internet searches for English words in construction for “construction stress”, “construction mental” and “construction suicide” with reference to “construction worker” has not previously been undertaken. It is important to understand internet interest across these 3 terms as all are relevant to mental health and stress in construction. Suicide risk is significantly complex and multifactorial. Our aim is to investigate internet search interest across construction with a focus on mental, stress and suicide, and determine whether there is consistent interest across these search terms. Methods: Using Google Trends, data on global search queries we compared “construction mental” or “construction stress” and “construction suicide”. Two time periods were compared, the last 5 years and the last 24 months, both till December 8th, 2022. The relationship between web search interest, reflected by search volume index (SVI) for society and community versus the business and industrial category and health category were evaluated. Results: Open category searches on Google trends for the key words “construction mental” or “construction stress” demonstrated moderate SVI peaks over a 5-year period. Sub-group analyses for the industrial and business category demonstrated consistent low interest in suicide compared to search terms related to stress or mental health. Conclusion: There is limited online interest in construction mental and stress and even less interest in suicide.

## Introduction

There is significant evidence in the literature to indicate that the construction industry faces an increased prevalence of workplace stress, mental illness, suicide ideation or suicide attempts, when compared to other industry sectors [1]. Decline in mental health in construction industry may be in part mitigated by reducing psychological and logistic stressors across work projects [1]. On the other hand, even if stress is reduced this may still not mitigate the need for suicide or mental health prevention strategies. For the individual worker, internal factors such as prior mental history, anhedonia, past trauma, and/ or external factors can influence individual suicide thoughts and attempts [2–4].

Previous studies have also shown that suicidal ideation in trainee construction workers is common and is associated with a reduced self-sense of belongingness [3]. Suicide risk can also be associated with addictive behaviours such as increases in substance use [3]. Among construction worker communities, learning of others who had attempted suicide or lost their life can increase suicide ideation and attempts [3]. This suggests that there is a role for construction industry in understanding risk factors and developing tailored workplace suicide prevention activities. Information for the construction industry on suicide can be sourced from the internet to inform workplace management of strategies and interventions to help understand and prevent suicide [5]. From an industry perspective the incentive to engage in prevention strategies would be based on economic costs of suicide in the workforce [6]. Indeed, there is an economic cost to the construction industry, particularly if there is an immediate network contagion effect on workforce productivity from each suicide attempt or death [7].

It has also been suggested that COVID may have increased stress in the construction industry with more projects and infrastructure investments to stimulate the economy during this period [1]. This suggests that there may be increased interest and/or need for construction information with respect to stress, mental health and suicide during the period of COVID [1]. With more projects there is also a recognized increase in stress that can affect cognitive attention, worker accidents and potential for worsening of mental health [1]. Stress in workers, if recognized and acted upon by managers allows for resilience and self-mitigation of factors contributing to workplace mental health decline [8]. Interestingly, it remains unknown whether there has been interest in Google searching for information regarding stress or mental problems in the construction industry.

The internet is a common source of online wellbeing information and solutions for industry and human resources. For example, we have previously shown an increase in resilience searches during COVID in the industrial and business category in Google trends [9]. Indeed, interest in online web searching for construction workers mental health, stress and suicide can be assessed by popular web search engines, such as Google [9]. Analysis of these trend data could allow for observing interest in search topics relevant to interventions or understanding factors that affect mental wellbeing in construction. The aims of this paper are to determine the role of searching of internet web browser traffic for construction worker or construction mental, construction stress and construction suicide.

## Methodology

### Google trends data availability

Google provides open-source application access to Google Trends, an infordemiology search tool for Google internet search volumes, encompassing global online keyword searchers and or topics over time and geolocation [10]. Google Trends has been increasingly utilized by industry to understand interest in worker mental health and or management strategies. Search activity data and graph outputs depict the Google search volume index (SVI), which is normalized over a specific time and or geographic location to improve comparability between search terms. The graphs are scaled on the y-axis over a range of 0–100, where 100 is the maximum search interest for the time and location specified. A score of 0 means there was not enough internet search data for this period or periods to reach above an arbitrary zero threshold.

### Search process and data retrieval

Online interest in construction workers analyzed using Google Trends. The keyword “construction worker” (as a reference SVI for construction) compared to “construction mental” or “construction stress” or “construction” was entered in the Google Trends main page (available at: http://www.google.com/trends, accessed Dec 8th 2022). We set the following filters for the analyzed queries: “Worldwide,” in “All categories” and compared to “business and industrial category” and “Health category” and restricted all internet traffic for “Web searches.” We collected SVIs on a 5 year and 2-year basis dating back from Dec 8th 2022.

### Data analysis

We qualitatively reported the changes over time of search queries with English speaking words and referred to English speaking countries where the SVI was significant to that country. We analysed SVI’s overtime that corresponded to COVID and non-COVID periods of time reported in Google Trends “all categories”. We also reviewed construction workers and compared the proportional search trends to construction mental; construction stress and construction suicide using these predefined terms, for all countries and two pre-defined search categories.

### Ethical considerations

This study involved aggregated non-identifiable key word internet google traffic. Hence, we are using summary open-source informatics data on google searches using Google trends. For this reason, the design of the study did not require Research Ethics approval.

## Results

**General category** Interest for all category search terms “construction worker” were compared to “construction mental” and “construction stress” and “construction suicide” for the last 5 years (globally). When compared to “construction worker” over 5 years there was a significant multi-fold decrease in average SVI for “construction mental” (see Table 1). Additionally, there was less SVI interest in “construction stress” and “construction suicide” compared to “construction worker” (Table 1). Only a handful of countries demonstrated an SVI in “construction mental” compared to other countries. Those countries where there was interest in construction mental included Australia, India, Canada, and United Kingdom. During the covid period from March 2020 to Dec 6th 2022, when “construction worker” SVI was compared to either “construction stress” or “construction mental” there was 30-fold less interest in these searches. Hence COVID did not increase the interest in these search terms.

**Table 1:** SVI for different search terms and google categories

	SVI’s across key search terms when compared to each other for 5 years			
Google Search Category	construction worker	construction stress	construction mental	construction suicide
General	57	3	2	1
Business industrial	34	2	1	1
Health	33	6	7	3

**Fig. 1:**
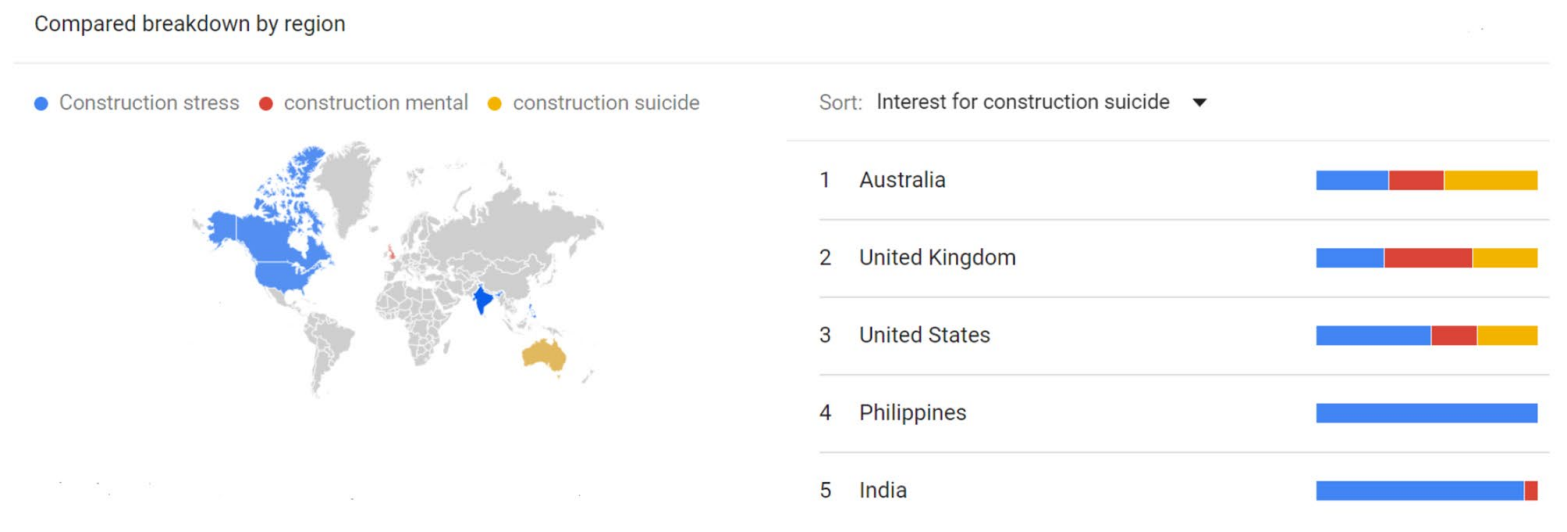
Country specific SVIs for “construction mental” “construction stress” construction suicide” and percent interest in the color bands for the industrial and business category

**Business and Industrial category** We next explored Google web trends category for the business and industrial. As in the previous all category search, SVI’s for “construction worker” were significantly higher when compared to “construction stress” or “construction mental” or construction “suicide”. We then explored which was more popular among SVI’s for “construction stress” “construction mental” and “construction suicide”. Specifically, we observed there was more than a doubling in the interest of “construction stress” when compared to the other two search terms. Both “construction mental” and “construction suicide” globally had the same average SVI’s over 5 years. When we compared globally which countries had a greater SVI interest in “construction suicide” compared to the other two terms, only Australia had a greater interest in suicide compared to mental or stress. And interestingly only 3 countries had an interest in all 3 search terms Australia, UK and USA (Fig. 1).

**Health category** In the health category there was a modest decline in SVI for “construction worker” compared to the other categories (Table 1). Compared to the search categories above (general and business industrial) there was a slight increase in SVI for “construction mental”, “construction suicide” and “construction stress” when compared to “construction worker” in the health category (Table 1). Additionally, while the SVI for “construction suicide” increased in this category compared to previous categories, however the proportional SVI remained the same when contrasted to “construction stress” and “construction mental”. A increase in searches around mental and stress in construction was observed, with further interest in suicide compared to other categories (see Table 1).

## Discussion

In analysing Google trends data using the key search terms “construction stress” and “construction mental” from the period of Dec 2017 to Dec 2022, we found that the worldwide average SVIs remained low over a period of 5 years. Searches for “construction suicide” were constantly lower in the general and industrial and business search categories compared to the other search terms. Additionally, the SVI’s for “construction stress” and “construction mental” were similar in two of the three search categories but higher in the health category.

We suggest that internet searches for construction stress may be associated as a proxy interest in search interest in mental health or suicide. Interestingly SVI’s for construction stress in the general category were limited to a few countries only and was more common in India and the Philippines as compared to Australia or the UK. This is surprising because suicide rates are known to be highest in the world in India, particularly across the agriculture sector and in the younger populations. It is also recognized that much of the Indian labor force is unorganized for construction work. Henceforth construction workers mental health and suicide risk may not be evaluated as part of the mobile supply of reduced cost labor [11]. Countries like Australia and the UK that have been proactive with awareness and intervention programs to reduce suicide risk in construction [12]. Possibly for this reason UK and Australia and Canada were unique to have had SVI’s that reached threshold compared to other countries for the search term “construction suicide” in the business and industrial category.

Numerous studies have alluded to the fact that there has been a global prevalence of persistent stress across various industries due to previous COVID lockdowns [8]. Whether global COVID internet search fatigue has influenced and maintained a reduced SVI related to mental and stress searches in construction remains unknown [13]. Pragmatically one would have expected to observe an increase in search interest during COVID for the word’s construction stress, construction mental and construction suicide. In the future it would be interesting to explore if media fatigue with COVID can account for the reduced SVI in the industry and business category for construction mental. On the other hand, we have shown previously increased Google searches for resilience in industrial and business categories and a reduced interest in wellness [8] during COVID. This may suggest that mental well-being interest is addressed by paying more attention to increasing industrial resilience. For example, it could be assumed that a more resilient industry sector may be less likely to be stressed, suffer mental illness, or have increased suicide risk [8].

In summary, COVID made little difference to the key word SVI’s for the general search category. As “construction worker” was the highest searched variable in the general category, we restricted SVIs for COVID to this category. Furthermore, the search term “construction worker” for the Google trends category business and industrial and general category mean SVIs were similar, yet both had a lower SVI than in the general category. This is interesting because it would be expected that there would have been greater internet interest in “construction worker” from the industrial and business category. Additionally, the SVI’s for “construction stress” and “construction mental” were similar in two of the three search categories but higher in the health category. In the general category we suggest that internet searches for construction stress may be associated with countries where there is interest in preventing mental health disorders or suicide.

To our knowledge, this is the first study analyzing worldwide English words in Google trends for construction themes related to mental, stress, and suicide. We have clearly identified a gap where there is more interest in construction workers compared to industry related stress, mental health, and suicide. Of significant concern is the very low interest in suicide for the construction industry globally, particularly across individual countries. It appears those countries where there is interest in suicide and mental health, have an active research and intervention strategy such as the UK and Australia [12].

This study may have some limitations, we acknowledge that Google is not the only web search engine, and terms were restricted to English language, hence the likely search results restricted to English speaking countries or where English is at least an official business language. We also note that the word “mental” was used as a generic term to encompass interest in both mental health and mental wellbeing. Because of the use of anonymous summary data, we cannot verify whether SVIs with construction are related to interventions, products, or information to support mental health outcomes in the construction industry sector. Given these limitations, we are cautious not to over interpret these results. We suggest the results may identify a potential gap for further investigation around the mental health landscape in construction. Particularly around the low interest in suicide, stress and mental health in the industry and business category for construction.

## Conclusions

Google Trends data analysis has shown that there was a reduced tendency to search for key terms, such as construction stress, construction mental or construction suicide in the business and industrial category. COVID has seen an increase in social economic structural reforms that have facilitated increased construction, however this did not translate to increased internet interest for mental searches in construction or the industry category. There were interesting observations arising from this study; firstly, construction suicide at a global level received less attention from the industry and business category compared to the health category, and secondly there were no proportional changes during covid for these key search terms and lastly, there was not an equal interest in both construction stress and construction mental searches across different English-speaking countries. Further research is required to ascertain whether there is enough balanced intertest in both mental health and suicide information and prevention based on the low SVI in business and industrial categories.

## Data Availability

Non applicable all data is available in the manuscript and freely online via google trends.
